# Deciphering fire tolerance of trees at the Amazonia–Cerrado transition by trait‐based approach: Implications from species to communities

**DOI:** 10.1002/ajb2.70066

**Published:** 2025-07-03

**Authors:** Wesley Jonatar A. Cruz, Manoela S. Machado, Francisco Navarro‐Rosales, Maria Antonia Carniello, Marcelo Leandro F. Andrade, Flávio C. Oliveira, Immaculada Oliveras Menor

**Affiliations:** ^1^ AMAP, Université de Montpellier, CIRAD, CNRS, INRAE, IRD Montpellier France; ^2^ Environmental Change Institute, School of Geography and the Environment University of Oxford Oxford UK; ^3^ Woodwell Climate Research Center MA USA; ^4^ Department of Biology University of Oxford Oxford UK; ^5^ Programa de Pós graduação em Ciências Ambientais (PPGCA), Universidade do Estado de Mato Grosso – UNEMAT, Campus de Cáceres MT Brazil; ^6^ ICMBio (Instituto Chico Mendes de Conservação da Biodiversidade) – Estação Ecológica da Serra das Araras MT Brazil

**Keywords:** ecophysiology, fire‐resistance, functional traits, mortality, novel fire regime

## Abstract

**Premise:**

Understanding how fire impacts trees is essential for predicting the effects of novel fire regimes on plant diversity in the transition between the world's two most diverse biomes, the Cerrado and the Amazonia. Here we addressed knowledge gaps regarding physiological damage and mortality in transitional species within fire‐prone ecosystems.

**Methods:**

In a manipulative fire experiment, we burned a transitional woodland savanna for six consecutive years after it had been fire‐excluded for 33 years. We classified the most abundant tree species according to their fire tolerance and examined the relationship between fire tolerance and key morphological and ecophysiological functional traits. These traits were related to leaf economics spectrum, bark investment, wood density, flammability, and physiological drought tolerance.

**Results:**

Species had three main fire tolerance strategies, reflected in their investment in the outer and inner bark, wood density in branches and the main trunk, changes in leaf water potential, and water and dry matter ratios in leaves. The inner and outer bark and the level of protection of the sprouting buds better explained tree mortality and topkill. Under very frequent fires, fire‐sensitive species had the highest mortality rates and fire‐thrivers became the most abundant species.

**Conclusions:**

Transitional tree species had different response strategies to fire based on their tolerance, which directly influences their survival and the overall structure of the community. Our findings suggest likely shifts in tree community structure in response to novel fire regimes.

## Graphical abstract

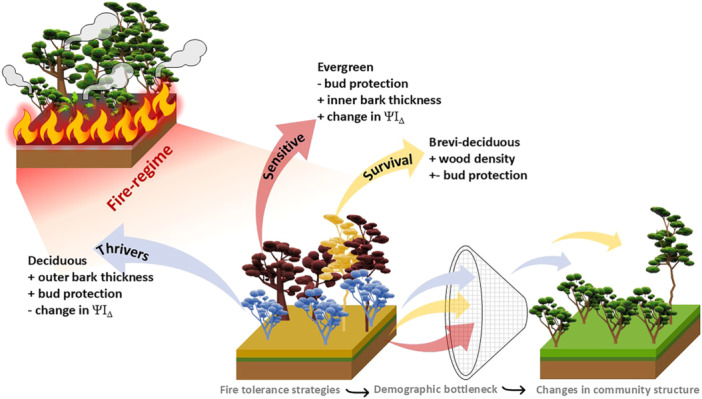

Transitional vegetation at the ecotone between the Amazonia and Cerrado biomes harbors a unique and complex set of Amazonian and savanna woody species of great biological and ecological importance (Torello‐Raventos et al., 2013; Marimon et al., 2014). This transitional vegetation is characterized by a mosaic of forests and savannas that undergo intense seasonal dry periods of 4 to 6 months, characterized by an absence of rain, high temperatures, and low humidity. As a result, plant species in this region are at their physiological limits and may be more sensitive to disturbance compared to woody species in the core areas of the Cerrado and Amazonia (Jancoski et al., 2022; Tavares et al., 2023). On the other hand, anthropogenic pressures are altering the fire frequency and intensity of the historical fire regimes at the transition zone (Pivello et al., 2021; Segura‐Garcia et al., 2024). Additionally, climate change is already impacting this region, driving weather conditions that promote more intense fires (Li et al., 2021). These abiotic drivers (shifts toward drier, hotter climates and changing fire regimes) can increase mortality rates, eliminating species lacking the morphofunctional or ecophysiological traits needed to survive these conditions (Scalon et al., 2020; Tavares et al., 2023).

The Amazonia–Cerrado transition is the ecotone between the fire‐dependent Cerrado and fire‐sensitive Amazonia. Fire plays a key role in shaping the structure and dynamics of these two components of the vegetation mosaic (Hoffmann et al., 2012; Oliveras and Malhi, 2016). In both forest and savanna environments, fire evolutionarily selects traits that enhance species persistence, leading to the coexistence of plants with different adaptive strategies. In the Cerrado, fire is ecologically significant in preventing woody encroachment and the thickening of savannas (Durigan and Ratter, 2016; Passos et al., 2018). It also functions as an alternative consumer of vegetation, maintaining open patches within the forest–savanna mosaic, as described by the black world species theory (Bond, 2005).

Although fires are evolutionarily and ecologically essential in most tropical ecosystems, their frequency has sharply increased. In 2022–2023, the Amazonia and Cerrado biomes were the two most affected by fire (number of events and area affected) compared to other biomes in South America (Projeto MapBiomas, 2023). Most of these fires were high intensity (high energy release) because they occurred during the peak of the dry season, causing high tree mortality and changes in soil structure, creating a novel fire regime. Because traditionally fires have occurred at the beginning of the rainy season in November, the vegetation is still dry and the first rains moisten the soil, favoring post‐fire regeneration and large fire control (Ramos‐Neto and Pivello, 2000; Silva et al., 2018). These novel fire regimes at the peak of the dry season devastate fire‐sensitive ecosystems (Amazonia) and also impact fire‐dependent ecosystems such as the Cerrado, changing vegetation composition and structure based on fire severity and frequency (Harvey and Enright, 2022).

Research on the vulnerability, resistance, and resilience to fire of tropical plant species is still limited (considering the diversity of life forms and environments), and the mechanisms behind fire survival are still far from being fully elucidated (West et al., 2016; Nolan et al., 2024). The central question in fire ecology is: “Why do some species die while others thrive after fire?” This question becomes even more complex when considering the wide range of morphofunctional and physiological traits among species in the same ecosystem.

Fire response strategies (mechanisms that the species use to persist in fire‐prone ecosystems; see Pausas, 2019) have been described through different fire‐related functional traits. These include the thermal insulation offered by the bark to the xylem and the phloem (Michaletz and Johnson, 2007); the trade‐offs and combination of bark thickness and density (Rosell et al., 2014); and wood size and density, which are important factors for determining tree mortality in forest species (Brando et al., 2012). Additionally, species can exhibit trade‐offs between investment in inner and outer bark (Scalon et al., 2020) and adaptations like bud protection below and above ground (Chiminazzo et al., 2021, 2023). The combination of these traits enables species to have different adaptive strategies to fire: fire‐resistance (i.e., immediate protection from fire without suffering damage); fire‐avoidance (avoiding contact or reducing the intensity of the fire); and fire‐tolerance (recovering from fire damage) (Pausas, 2019). Fire‐related traits play a central role in explaining the persistence and recruitment of trees in the fire zone (impacted by flames) for years, after subsequent burning events (Gignoux et al., 2009), and demographic bottlenecks characterized by tree mortality in woodland savannas (Hoffmann et al., 2012).

A trait‐based approach is valuable for understanding the mechanistic underpinnings of fire response strategies and their relationship with species fitness, because it accounts for the complex interactions among various functional traits (Clarke et al., 2013; Rosell et al., 2014; Scalon et al., 2020). This approach can help predict how species in fire‐prone ecosystems will respond to drastic changes in fire regimes (e.g., high frequency, total exclusion of fire, and high intense fires) (Osborne et al., 2018). Studying plant strategies in the Amazonia–Cerrado transition using a trait‐based approach will help address gaps in our understanding of how species cope with fire and the vulnerability of woody species in this ecotonal area. Given the vulnerability of plant communities in the Amazonia–Cerrado transition—shaped by the intricate interplay of climatic, physiological, and anthropogenic factors—this region presents a unique opportunity to investigate how tree species with different levels of fire tolerance respond to fire disturbances, particularly in light of novel fire regime.

Here we investigated the relationship between functional traits and post‐fire response tolerance of tree species and their impact on community dynamics (Figure 1). We selected 15 target tree species that are representative in terms of frequency, abundance, and ecology of the plant communities at the Amazonia–Cerrado transition (Reis et al., 2015). For these species, we assessed the response traits relevant to the main ecological strategies, including the leaf economic spectrum (e.g., specific leaf area, leaf dry matter content, and leaf thickness; see Wright et al., 2004), bark investment (Rosell et al., 2014), wood density (Brando et al., 2012), flammability (Pérez‐Harguindeguy et al., 2016) and physiological drought tolerance (Wigley et al., 2021). Using an experimental approach, we measured tree mortality rates, and through a short‐term experiment, we assessed topkill and indirect effects (e.g., physiological changes; see Michaletz and Johnson, 2007) with data collected before and after fire. We then employed a trait‐based analysis to classify species according to their fire response strategies, guiding us in addressing the following questions.

(1) What are the fire response strategies of the most abundant tree species at the Amazonia‐Cerrado transition? We hypothesized that the main fire response strategies will be fire avoidance (investment in resource acquisition and growth; Lawes et al., 2011; Scalon et al., 2020) and fire resistance (investment in protection traits aimed at plant defense such as bark thickness, leaf thickness, bud protection). We also expected that some species would have intermediate avoid‐resistance strategies (e.g., investment in growth and bark) considering that this is a floristically transitional community (Morandi et al., 2016).
(2) Which traits best explain post‐fire tree survival? Based on previous research on woody species (Lawes et al., 2011; Brando et al., 2012; Charles‐Dominique et al., 2015; Scalon et al., 2020; Chiminazzo et al., 2023), we hypothesized that post‐fire survival is strongly related to investment in outer bark, which provides primary protection against fire, and the level of bud protection, which enables post‐fire recovery, even in cases of topkill. Additionally, we expected that fire damage would result in more negative water potentials due to damage to the xylem and cambium (Bär et al., 2019), contributing to tree mortality in transitional savannas.
(3) What are the implications of fire tolerance for the structure of tree communities in the Amazonia–Cerrado transition? We expected that fire‐sensitive species, which are naturally abundant, will experience high mortality, allowing more fire‐tolerant, less‐abundant species to proliferate, resulting in a demographic shift and a reduction in species diversity. The profound impact of fire disturbances on savanna structure and composition is likely to manifest through a demographic bottleneck, characterized by high mortality of the woody component (Hoffmann et al., 2012).

**Figure 1:**
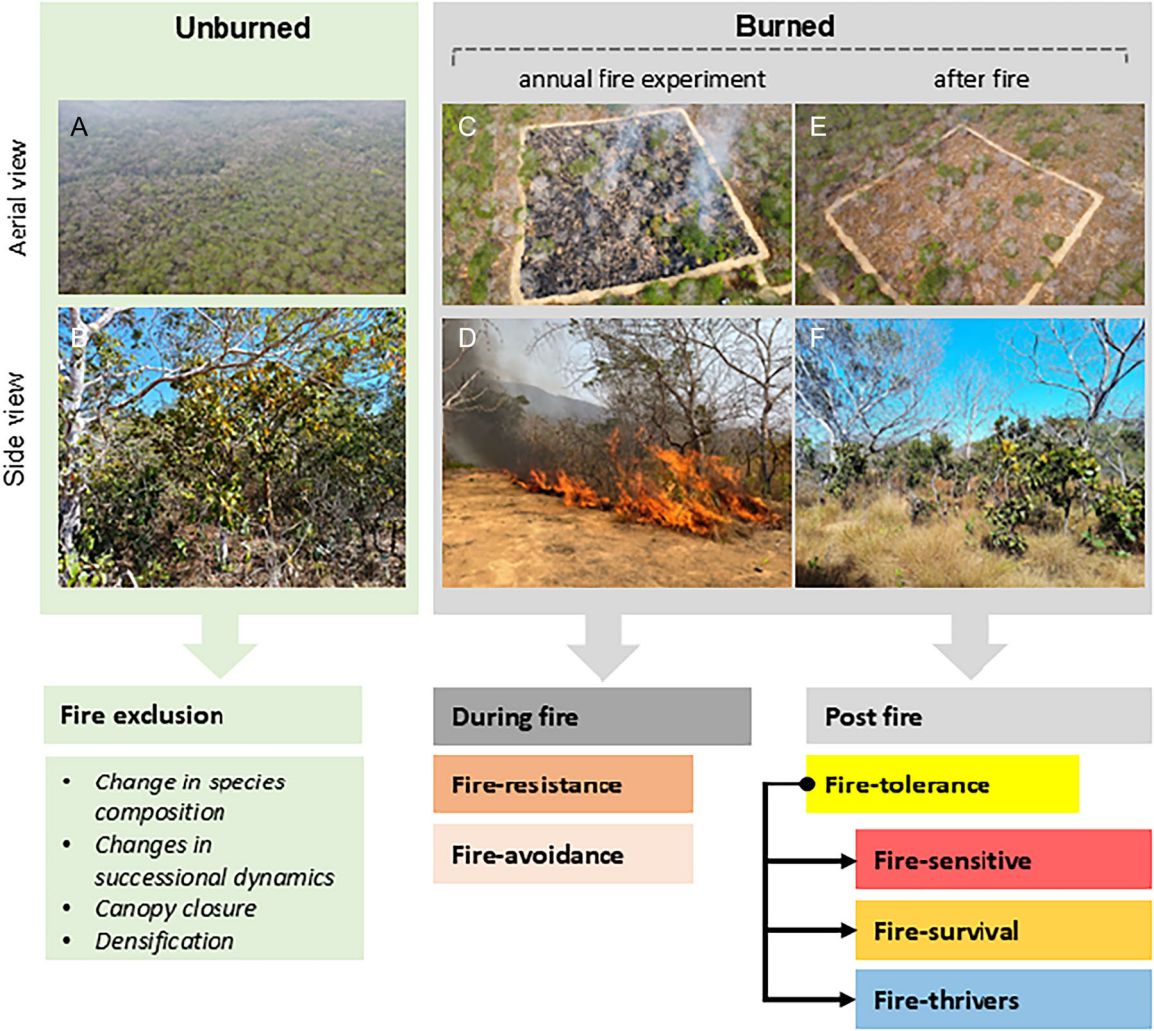
Transitional savanna unburned under a fire exclusion regime (A, B) highlighting the densification of the vegetation, record of an experimental burn in 2024 on the plot, which was burned every year since 2018 (C, D); transitional savanna after six experimental burn events (2018–2023) showing a more open ecosystem, tree mortality and different fire‐tolerance strategies (E and F).

## MATERIALS AND METHODS

### Study area

Our study site is located in a transitional savanna (typical woodland savanna) in the ecotone between the Amazonia and Cerrado biomes in the Serra das Araras Ecological Station (15°39′10.96″ S, 057°12′52.54″ W). The area has two well‐defined seasons, one characterized by a strong drought from May to September (total rainfall during this period <150 mm) and a rainy season from October to April, when rainfall is concentrated (1200–1300 mm). Minimum temperatures are between 18° and 23°C, and maximum temperatures can be between 30° and 42°C (Fick and Hijmans, 2017). No wildfires have been recorded in the area since 1992.

Within the study area, we used data from two distinct field sampling set ups to better measure plant functional traits and analyse the fire sensitivity of Cerrado tree species. First, we relied on a pair of monitoring plots subjected to a long‐term fire experiment to obtain reliable community‐scale data on species abundance, mortality, and fire tolerance, meaning we were able to capture fire effects in broad temporal and spatial scales. The second measurement area consisted of a 10 × 100 m transect where plant functional traits and topkill were evaluated, allowing for more intensive sampling and detailed monitoring of short‐term fire impacts in relation to the overall community effects.

### Abundance and mortality rate data

To access the mortality rate and abundance for each species, we used data from inventories in two 1‐ha plots, one burned annually since 2018 (i.e., six times, Figure 1C–F) and one that had not burned since 1992 (unburned, Figure 1A, B). Both plots were inventoried every 2 years starting in 2017 (2017, 2019, 2021, 2023). Fires were carried out at the peak of the dry season (July–September) to simulate an altered fire regime in the Cerrado (Pivello et al., 2021), intensified by the extreme conditions of the dry season (low relative humidity and high daily temperatures). All trees with DBH ≥ 5 cm in both 1‐ha plots were measured and identified at the species level. These plots are re‐censured every 2 years and monitored for primary productivity dynamics (Navarro‐Rosales et al., 2025), but are not destructively sampled in order to maintain natural community dynamics. We classified the families according to APG IV (Chase et al., 2016) and revised species names using the Flora e Funga do Brazil (2025). Botanical specimens were deposited in the permanent collection of the Herbário do Pantanal Vali Joana Pott (HPAN; Universidade do Estado de Mato Grosso, Cáceres, MT, Brazil).

Importantly, the experimentally burned area is of sufficient size (100 m) to ignite a fire line that allows a self‐sustained fire that imitates initial wildfire conditions. The fire line was set at 10 m from the plot so it could reach some self‐sustained spread when reaching the plot (Figure 1C, D). We recognize that 1‐ha plots divided into 25 subplots are a necessary form of pseudoreplication and are often associated with experimental fires (Balch et al., 2008). There are no plot replicates in the experimental fire because each fire is unique in terms of behavior and spread, and disturbance events such as large‐scale wildfires are impossible to replicate or randomize (van Mantgem et al., 2001).

### Fire tolerance of tree species

We categorized the species according to their fire tolerance using an adaptation of the empirical classification developed by Oliveras et al. (2014), fire tolerance index (FTI), combined with the mortality rate (MR) in the burned plot: FTI = No.Ind_burnt plot_ – No.Ind_unburnt plot_/No.Ind_unburnt plot_, where No.Ind is the number of individuals.

We categorized the species as fire‐sensitive when FTI ≤ –0.5 and MR > 20, (i.e., species strongly declined in numbers of individuals and high mortality rates in burnt plots), as fire‐survivors when FTI > −0.5, ≤ 0.5 and MR ≥ 5, ≤ 20 (i.e., species had subtle changes in numbers of individuals and moderate mortality rate), or as fire‐thrivers when FTI > 0.5 and MR < 5 (i.e., species became abundant and mortality rate was low in burned plots).

### Tree topkill and functional trait data

To represent the functional structure of the community in detail and account for short‐term responses in fire, we established a second experimental area (10 × 100 m transect) adjacent to the annually burned plot. In this transect, we selected the most‐abundant species that contributed up to 80% of the basal area on the burned plot after the fire. For each species, we selected five individuals (≥5 cm DBH). Functional traits were measured between September 2022 and December 2023. Then the transect was burned in 2023 at the same time as the annually burned 1‐ha plot. In the transect, all individuals were monitored twice a month up to 3 months after the fire and assessed for the presence of topkill and leaf water potential after the fire. An individual was considered topkilled when its aboveground biomass was dead but presented basal and/or underground resprouting.

For each selected individual, we collected five leaves, a twig ≥1 cm in diameter, and a 2‐cm^2^ bark sample 30 cm from the ground. We measured and calculated specific leaf area (SLA) as the ratio of fresh leaf area to dry leaf mass after 60 h in an oven at 65°C; leaf dry matter content (LDMC) and leaf water content (LWC) as the ratio of saturated mass to dry leaf mass; leaf thickness (LT), outer bark thickness (OBT), and inner bark thickness (IBT) with a digital caliper (precision 0.001 mm); bark density (BD), stem wood density (SWD) and twig wood density (TWD) by the ratio of fresh volume to dry mass (Rosell et al., 2014); we measured leaf water potential (*Ψ*_I_) in predawn and midday of three leaves per individual using a pressure chamber (PMS Instruments Co.; Scholander et al., 1965). We used as maximum leaf water potential (*Ψ*_IMAX_) the most negative value of *Ψ*_I_ obtained in the dry season, and for change in leaf water potential (*Ψ*_IΔ_) we calculated the difference between *Ψ*_I_ measured before and after the fire.

In the twigs, we classified bud protection (BP) using a longitudinal cut according to the method of Burrows et al. (2010) and Charles‐Dominique et al. (2015): p0, completely exposed buds; p1, emerging buds protected by the meristem; p2, buds located inside depressions in the bark; p3, buds completely protected below the bark. We used the available literature (Pirani and Pedroni, 2009; Silvério and Lenza, 2010; Scalon et al., 2020) to classify each species according to its vegetative phenology (PHE): evergreen (EG), brevi‐deciduous (BD), deciduous (DE).

### Analysis

To understand the fire‐response strategies that trees use to deal with fire (question 1) and the role that traits play in fire survival, we first sought to understand the covariance in the trait space. Using all the traits, we carried out a principal component analysis (PCA) using the R packages FactoMineR (Lê et al., 2008) and factoextra 1.0.7 (Kassamba and Mundt, 2020). Finally, we compared the difference in the distribution of the traits between the fire‐tolerance groups using the Kruskall–Wallis test with post hoc Wilcoxon test and Bonferroni correction.

To investigate which functional trait best explains the topkill and mortality rate (question 2), we calculated the mortality rate (MR) of each species in the plot burned annually between the years 2017 and 2023:

$$\text{MR}=\left[1-{\left(\frac{\text{Nf}}{\text{Ni}}\right)}^{\frac{1}{t}}\right]\times 100$$

where Ni is the initial number of living trees in the 2017 census, Nf is the number of living trees at the end of the 2023 census, and *t* is the time period (in years) over which mortality is measured.

We then used a random forest analysis to assess the set of traits that better explain the mortality rate and topkill using the R package randomForest (Liaw and Wiener, 2002). Variable selection is based on the variable importance score (VIS), which is calculated by considering the increase in the mean square error (i.e., out‐of‐bag error, OOB) of a tree in the forest when the observed values of a variable are permuted in OOB samples (Genuer et al., 2010). Variable selection is carried out in three stages: (1) Irrelevant variables are eliminated; (2) all variables that are related to the response variable are selected, and (3) the selection is refined by eliminating variable redundancy (Genuer et al., 2010, 2015).

To assess the implications of fire tolerance of tree species on community structure (question 3), we built a mixed linear model to test whether there is a difference in the change in abundance over time (final abundance‐initial abundance = *Δ*_abundance_) between species in the plot with annual fire treatment (burned) and control (unburned). In this model, we considered *Δ*_abundance_ as the response variable, plot as a fixed effect predictor variable, and species as a random effect variable interacting with the plot for model = lmer(delta~plot + (plot|species)). In addition, we constructed dominance curves (Whittaker diagrams) to illustrate the change in the dominance ranking of tree species in the presence of the effect of fire through the mortality (or not) of sensitive species. Nonmetric multidimensional scaling (NMDS) was conducted to ordinate tree species and fire treatment. For species composition, we used Bray–Curtis distance based on species abundance data. All analyses were carried out using R version 4.3.3 (R Core Team, 2024) and statistical differences were considered significant when *P* < 0.05.

## RESULTS

### Identifying fire response strategies

**Figure 2:**
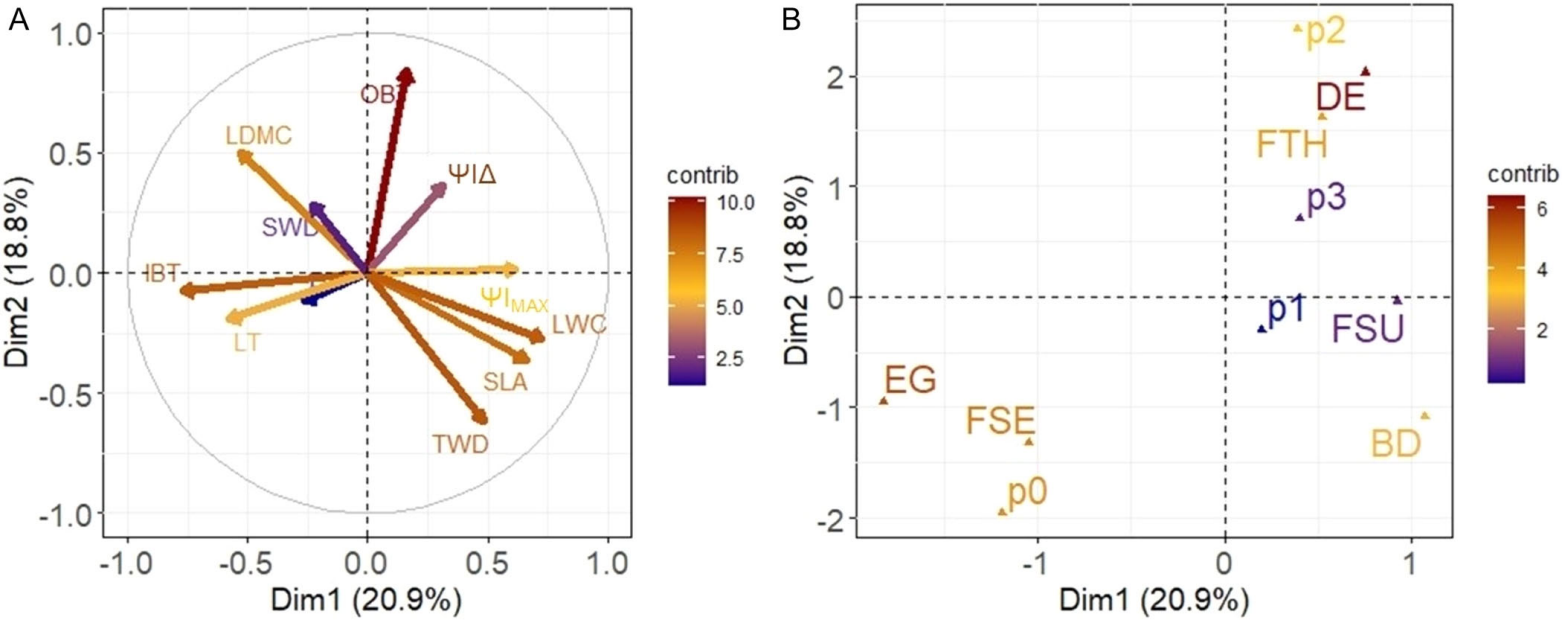
PCA results for transitional tree species showing distribution of vectors of (A) quantitative and (B) qualitative functional traits for transitional savanna tree species. SLA = specific leaf area, LDMC = leaf dry matter content, LT = leaf thickness, LWC = leaf water content, *Ψ*_IMAX_ = maximum leaf water potential, *Ψ*_IΔ_ = change in leaf water potential, OBT = outer bark thickness, IBT = inner bark thickness, BD = bark density, SWD = stem wood density, TWD = twig wood density, EG = evergreen, BD = brevi‐deciduous, DE = deciduous, FSE = fire‐sensitive; FSU = fire‐survival, FTH = fire‐thrivers, p0‐p3 = bud protection levels.

**Figure 3:**
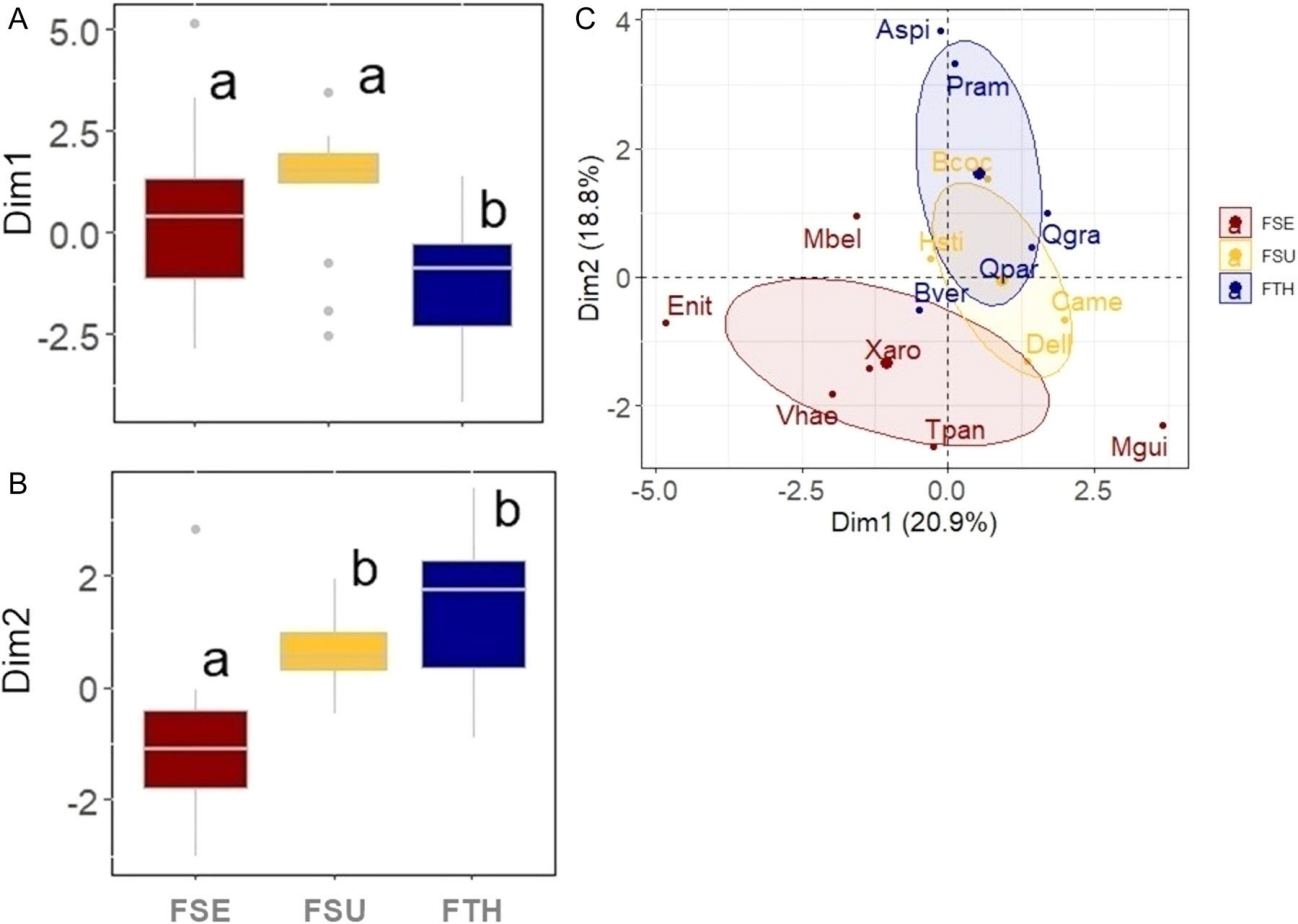
PCA results for transitional tree species showing a comparison of the scores for axis (A) 1 and (B) 2 between the fire tolerance groups; (C) distribution of species groups considering their fire tolerance. FSE = fire‐sensitive; FSU = fire‐survival, FTH = fire‐thrivers. *Species codes are defined in Table 1.

We assessed functional and ecophysiological traits, topkill, and mortality rates of key tree species classified according to their fire tolerance in a transitional savanna (Table 1). The first axis of the PCA (Dim1) explained 20.9% of the total variation in the functional space, primarily capturing the leaf economic spectrum and acquisitive strategies. This axis was strongly associated with leaf water content (LWC), specific leaf area (SLA), leaf dry matter content (LDMC), inner bark thickness (IBT), twig wood density (TWD), and maximum leaf water potential (*Ψ*_IMAX_) (Figures 2, 3; Appendix S1). The second axis (Dim 2), accounting for 18.8% of the variation, represented more conservative and fire‐resistance strategies, with phenology (PHE), outer bark thickness (OBT), and bud protection (BP) as the key correlated traits (Figures 2, 3; Appendix S1). Fire‐sensitive species (FSE) and fire‐thriving species (FTH) were best distinguished along the second axis of the PCA, with FSE species clustering in the negative region of Dim 2 (Figure 3). Additionally, fire‐sensitive species had higher scores on Dim1 (*Χ* ^2^ = 13.91, df = 2, *P* = 0.001, Figure 3), while fire‐thriving species scored higher on Dim2 (*Χ* ^2^ = 22.51, df = 2, *P* = <0.001, Figure 3). With regard to the leaf economic spectrum and acquisitive strategies (Dim1), FSE species were similar to FSU investing more in this strategy, different from FTH. For the more‐conservative strategies related to fire‐resistance (Dim2), FSU and FTH were similar and invest more compared to the FSE (Figure 3). The relationship between tolerance strategies and the axes based on traits confirming FSU as an intermediate tolerance category that shares strategies with FSE and FTH.

**Table 1:** Topkill, mortality rate, and functional traits mean (and standard deviation) of tree species in a transitional savanna.

Species	Code	Topkill (%)	Mortality rate (%year^–1^)	SLA (mm^2^ mg^–1^)	LDMC (%)	LT (mm)	LWC (%)	*Ψ*_IMAX_ (MPa)	*Ψ*_IΔ_ (MPa)	OBT (mm)	IBT (mm)	BD (g cm^–3^)	SWD (g cm^–3^)	TWD (g cm^–3^)	PHE	BP
**Fire‐thrivers**																
*Aspidosperma* sp.	Aspi	0	0	3.51 (0.30)	40.05 (2.20)	0.25 (0.02)	59.95 (3.32)	–5.03 (1.02)	–0.44 (1.32)	15.34 (0.74)	5.87 (1.1)	0.2 (0.15)	0.74 (0.12)	0.37 (0.06)	DE	p3
*Byrsonima verbascifolia* (L.) DC.	Bver	0	0	5.26 (0.58)	50.86 (3.41)	0.29 (0.04)	49.14 (3.41)	–3.23 (1.02)	–1.74 (1.16)	3.35 (0.85)	5.33 (1.8)	0.35 (0.03)	0.29 (0.02)	0.4 (0.05)	EG	p1
*Pouteria ramiflora* (Mart.) Radlk.	Pram	1.33	1	6.67 (2.7)	53.1 (6.1)	0.24 (0.01)	46.9 (6.1)	–3.02 (1.04)	–2.25 (1.29)	13.76 (5.17)	5.2 (1.1)	0.56 (0.16)	0.4 (0.03)	0.38 (0.04)	DE	p2
*Qualea grandiflora* Mart.	Qgra	1.33	2	6.49 (1.36)	53.02 (5.99)	0.3 (0.08)	46.98 (5.99)	–3.14 (1.06)	–0.92 (0.71)	7.88 (1.07)	2.77 (0.57)	0.5 (0.05)	0.41 (0.03)	0.46 (0.06)	DE	p1
*Qualea parviflora* Mart.	Qpar	2.66	0	7.4 (2.15)	41.91 (2.46)	0.32 (0.05)	58.09 (2.46)	–3.35 (1.17)	–1.96 (1.42)	9.45 (3.34)	3.56 (1.34)	0.48 (0.03)	0.41 (0.02)	0.46 (0.06)	DE	p1
**Fire‐survivors**																
*Byrsonima coccolobifolia* Kunth	Bcoc	0	3	7.14 (1.8)	52.72 (0.19)	0.28 (0.04)	47.24 (0.19)	–3.58 (0.82)	–1.52 (0.02)	9.37 (3.33)	5.9 (1.8)	0.45 (0.04)	0.31 (0.002)	0.37 (0.01)	DE	p2
*Curatella americana* L.	Came	0	5	7.85 (1.1)	42.62 (4.26)	0.29 (0.04)	57.38 (4.26)	–2.76 (1.04)	–1.45 (1.2)	6.33 (2.28)	5.44 (1.4)	0.38 (0.07)	0.48 (0.01)	0.53 (0.01)	BD	p3
*Davilla elliptica* A.St.‐Hil.	Dell	1.33	18	7.89 (0.56)	44.9 (2.36)	0.36 (0.03)	55.1 (2.36)	–3.59 (1.21)	–2.59 (1.48)	6.48 (5.85)	3.69 (1.0)	0.45 (0.09)	0.48 (0.01)	0.53 (0.01)	BD	p3
*Hymenaea stigonocarpa* Mart. ex Hayne	Hsti	0	8	6.48 (0.46)	54.47 (2.20)	0.21 (0.12)	45.53 (1.06)	–4.16 (1.02)	–3.5 (1.14)	8.65 (2.35)	5.99 (1.70)	0.29 (0.12)	0.79 (0.16)	0.38 (0.12)	BD	p1
**Fire‐sensitive**																
*Emmotum nitens* (Benth.) Miers	Enit	4	24	4.27 (0.52)	56.68 (2.92)	0.4 (0.12)	43.32 (2.32)	–3.97 (0.37)	–2.41 (0.59)	1.3 (0.71)	7.41 (1.26)	0.47 (0.19)	0.73 (0.12)	0.35 (0.04)	RG	p1
*Maprounea guianensis* Aubl.	Mgui	6.66	100	25.71 (5.12)	28.57 (3.23)	0.16 (0.9)	71.43 (4.10)	–4.49 (1.02)	–1.22 (1.25)	2.5 (1.2)	4.28 (1.02)	0.18 (0.05)	0.55 (0.01)	0.48 (0.06)	BD	p1
*Myrcia bella* Cambess.	Mbel	1.33	29	5.95 (0.81)	55.68 (2.25)	0.32 (0.02)	44.32 (2.25)	–3.68 (0.97)	–1.13 (0.39)	11.28 (1.05)	5.61 (1.76)	0.3 (0.1)	0.8 (0.21)	0.39 (0.02)	EG	p3
*Tachigali paniculata* Aubl.	Tpan	1.33	34	7.71 (1.39)	46.5 (1.76)	0.3 (0.05)	53.5 (3.69)	–2.98 (1.26)	–2.47 (0.91)	6.47 (3.41)	5.75 (1.36)	0.5 (0.1)	0.34 (0.21)	0.58 (0.08)	EG	p0
*Vochysia haenkeana* Mart.	Vhae	4	26	5.01 (0.47)	50.27 (1.76)	0.32 (0.03)	49.73 (1.76)	–3.22 (1.08)	–1.9 (1.63)	3.85 (3.47)	6.56 (2.38)	0.49 (0.05)	0.33 (0.06)	0.47 (0.06)	EG	p0
*Xylopia aromatica* (Lam.) Mart.	Xaro	6.66	76	10.94 (3.17)	44.21 (4.88)	0.33 (0.07)	55.79 (4.88)	–3.17 (0.88)	–2.94 (1.01)	5.92 (1.46)	6.56 (2.38)	0.46 (0.12)	0.29 (0.01)	0.40 (0.03)	BD	p0

Abbreviations: BD = bark density, BP = bud protection, IBT = inner bark thickness, LDMC = leaf dry matter content, LT = leaf thickness, LWC = leaf water content, OBT = outer bark thickness, PHE = vegetative phenology, SLA = specific leaf area, SWD = stem wood density, TWD = twig wood density, *Ψ*_IMAX_ = maximum leaf water potential, *Ψ*_IΔ_ = change in leaf water potential.

**Figure 4:**
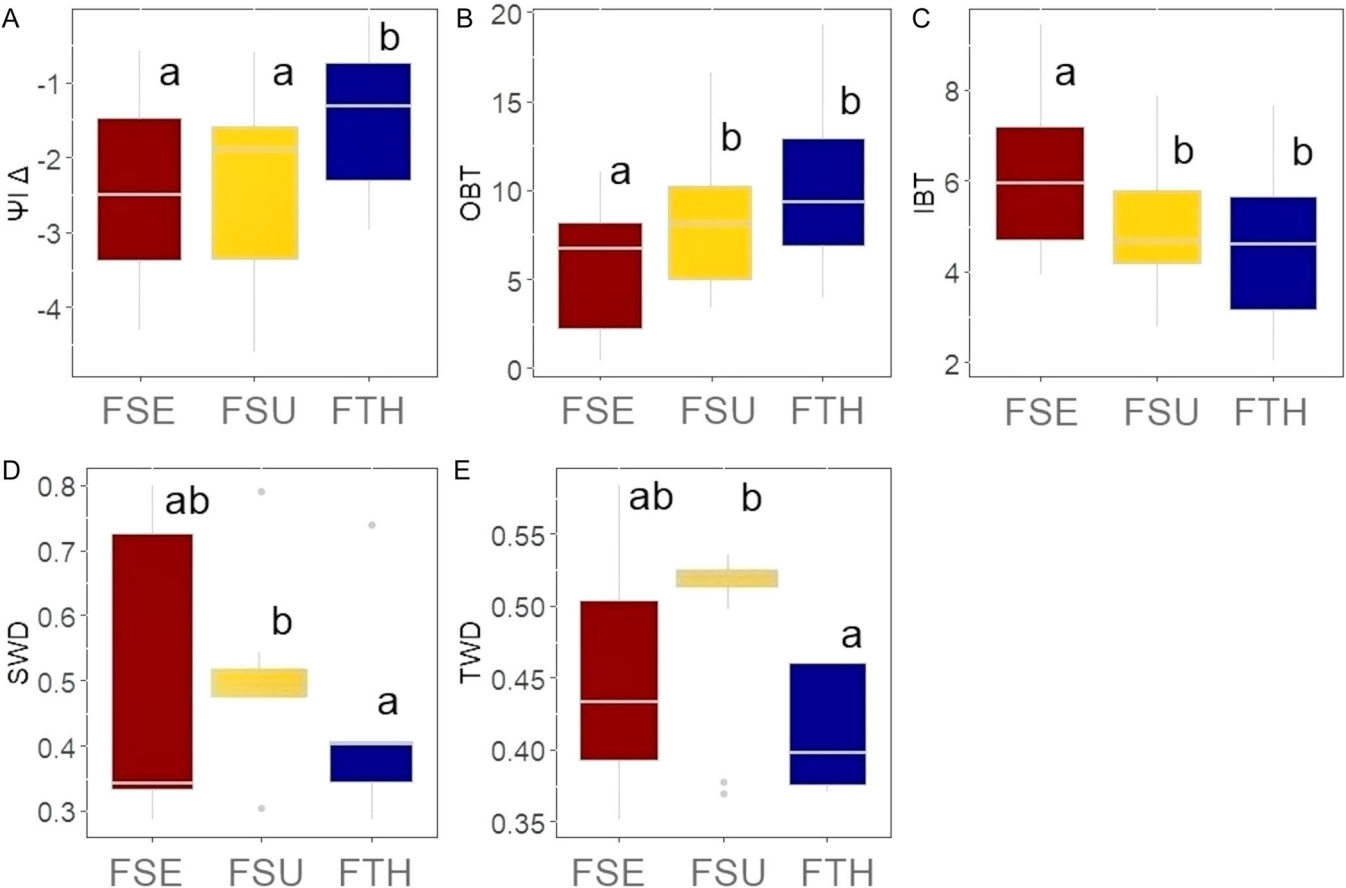
Comparison of functional traits between different fire tolerance strategies in transitional savanna tree species. (A) *Ψ*_IΔ_ = change in leaf water potential (MPa); (B) OBT = outer bark thickness (mm); (C) IBT = inner bark thickness (mm); (D) SWD = stem wood density (g cm^–3^); (E) TWD = twig wood density (g cm^–3^); FSE = fire‐sensitive; FSU = fire‐survival; FTH = fire‐thrivers. Different letters denote a significant difference in Kruskal–Wallis test (*P* < 0.05). Comparisons for remaining traits that were not significant are in Appendix S2: Figure S5.

Among the different strategy groups, fire‐sensitive (FSE) and fire‐survival (FSU) species had a greater change in leaf water potential (*Ψ*_IΔ_) compared to fire‐thriving (FTH) species (*Χ* ^2^ = 3.90, df = 2, *P* = 0.014; Figure 4; Appendix S2: Table S2). Fire‐sensitive (FSE) species had lower outer bark thickness (OBT, *Χ* ^2^ = 4.20, df = 2, *P* = 0.012; Figure 4; Appendix S2: Table S2) and higher inner bark thickness (IBT, *Χ* ^2^ = 10.30, df = 2, *P* = 0.005) than both fire‐survivor and fire‐thriving species (Figure 4; Appendix S2: Table S2). Wood densities in the stem (SWD; *Χ* ^2^ = 6.28, df = 2, *P* = 0.043, Figure 4; Appendix S2: Table S2) and branch (TWD; *Χ* ^2^ = 8.27, df = 2, *P* = 0.015, Figure 4) were higher in the fire‐survivor species (Figure 4; Appendix S2: Table S2).

### Functional traits influencing topkill and post‐fire mortality

As expected, the random forest models identified OBT, IBT, and BP as key variables explaining patterns of topkill and mortality rate (Table 2). Additionally, topkill was strongly associated with LDMC and LWC, and mortality rate was most closely linked to LT (leaf thickness) (Table 2).

**Table 2:** Variable importance score of the random forest models generated for each variable selection: Topkill and mortality rate.

	Variable importance score	
Trait	Topkill	Mortality rate
SLA (mm^2^ mg^–1^)	1.80	–0.43
LDMC (%)	**4.43**	–5.64
LT (mm)	–0.44	**10.84**
LWC (%)	**3.54**	–6.56
*Ψ*_IMAX_ (MPa)	0.51	–6.58
*Ψ*_IΔ_ (MPa)	2.32	–7.91
OBT (mm)	**3.40**	**18.81**
IBT (mm)	**6.59**	**25.38**
BD (g cm^–3^)	0.41	–26.41
SWD (g cm^–3^)	1.22	–34.97
TWD (g cm^–3^)	2.60	–6.56
PHE	2.55	–3.07
BP	**2.66**	**26.80**

Abbreviations: BD = bark density, BP = bud protection, IBT = inner bark thickness, LDMC = leaf dry matter content, LT = leaf thickness, LWC = leaf water content, OBT = outer bark thickness, PHE = vegetative phenology, SLA = specific leaf area, SWD = stem wood density, TWD = twig wood density, *Ψ*_IMAX_ = maximum leaf water potential, *Ψ*_IΔ_ = change in leaf water potential.

Selected variables are shown in bold.

### Effects of fire on community structure

**Figure 5:**
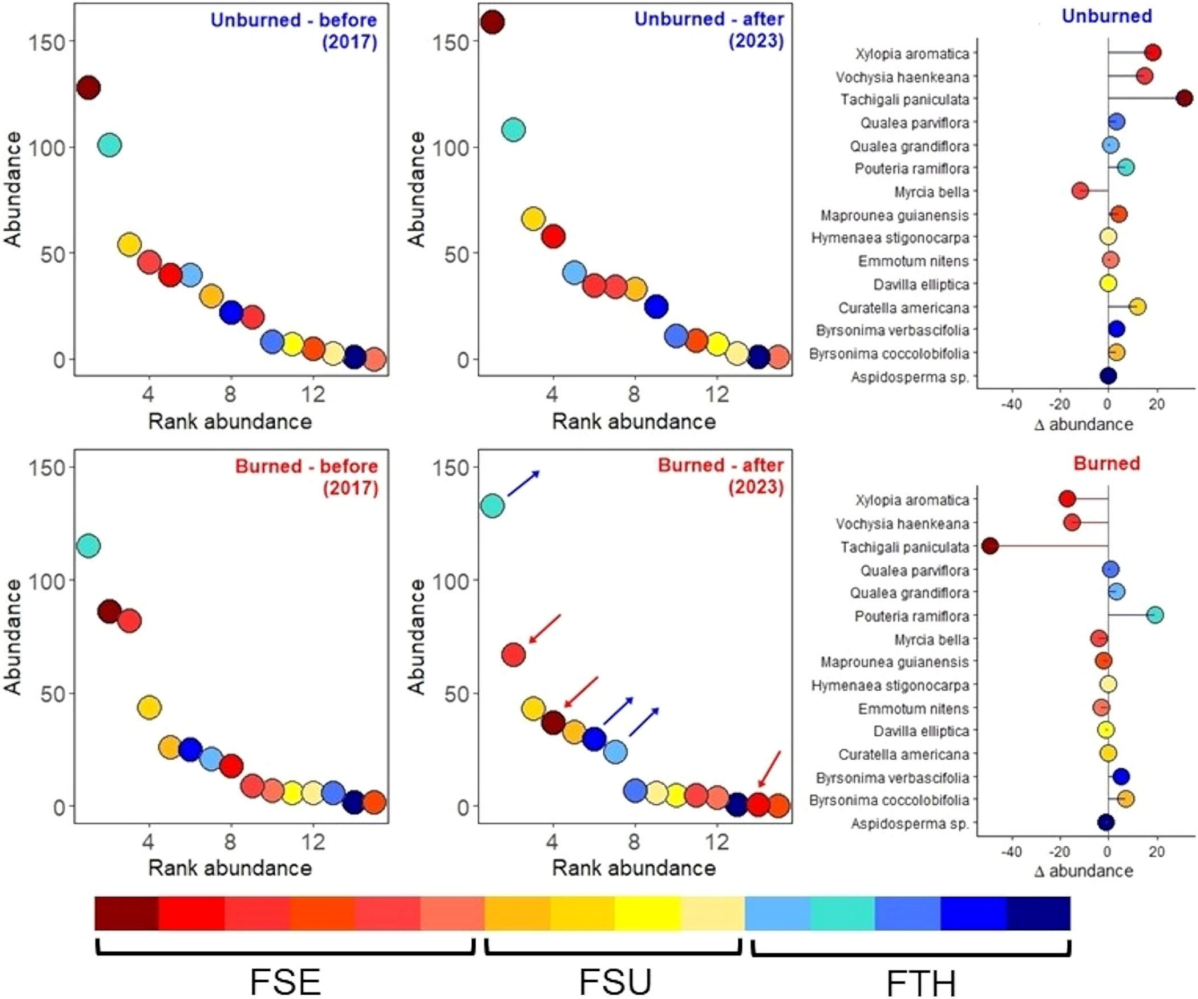
Dominance curves for tree species in transitional savanna in unburned (top row) and burned (bottom row) plots before (2017) and after (2023). The graphs on the right illustrate the coefficients of change in abundance before and after. Arrows indicate species with a marked reduction (red) and increase (blue) in the number of individuals. Color scale for fire tolerance strategy of species: red, fire‐sensitive (FSE); yellow, fire‐survival (FSU); blue, fire‐thrivers (FTH).

**Figure 6:**
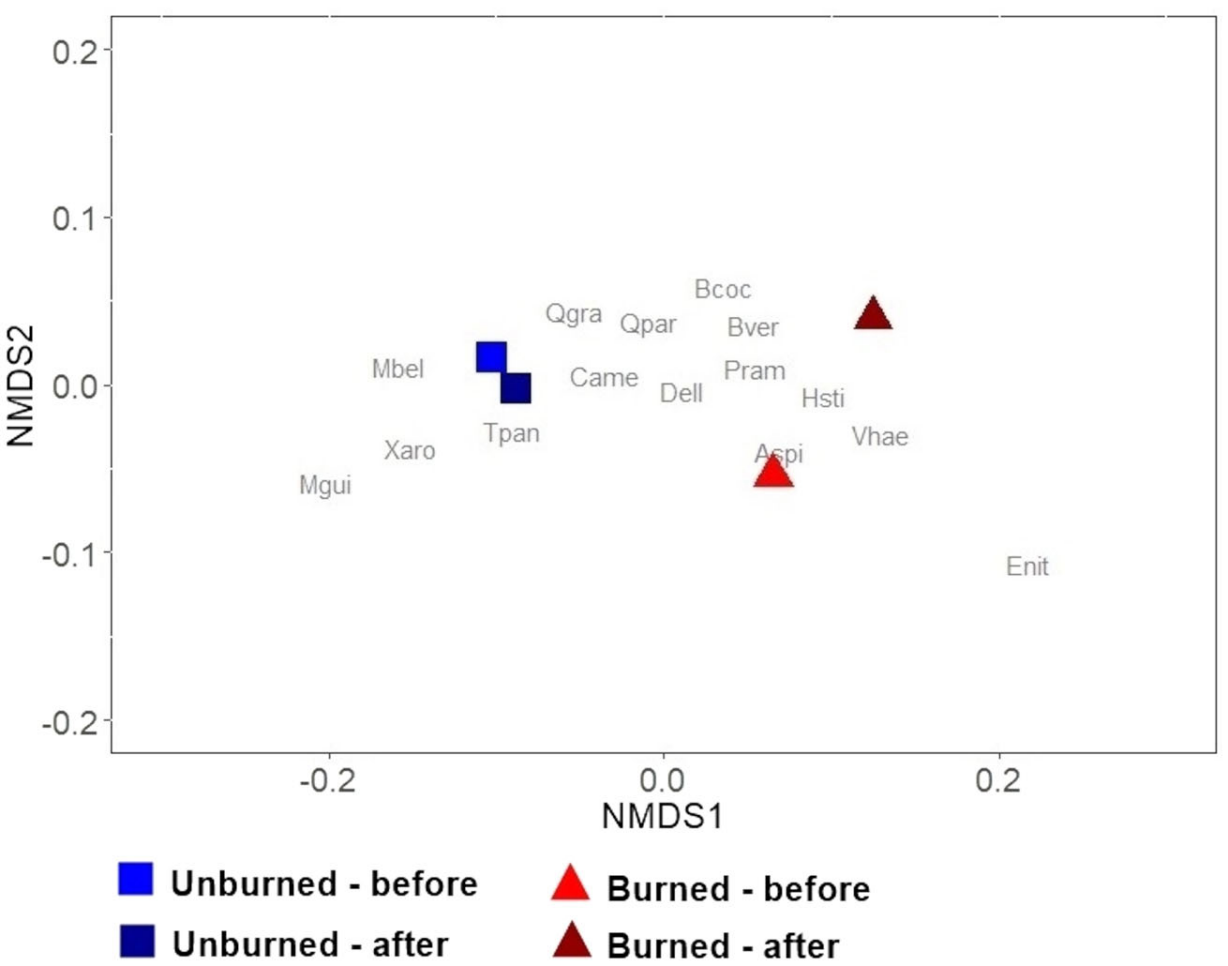
NMDS showing the distribution of burned and unburned plots according to tree species abundance before (2017) and after (2023) the burning treatment in transitional savanna.

A comparison of changes in species abundance between the beginning and end of the burning experiment and the unburned (fire exclusion) area revealed key changes in the distribution of species abundance, mainly in the relationship between dominant and subordinate species (*F* = 2.51, df = 14, *P* < 0.05; Figure 5; Appendix S3). Fire‐sensitive species such as *T. paniculata*, *V. haenkeana*, and *X. aromatica* substantially increased in abundance in the unburned area (respective *Δ*_abundance_ = 29.74, 13.68, 15.99) but sharply declined in the fire treatment area (*Δ*_abundance_ = 46.35, –14.82, –17). Conversely, fire‐thrivers species such as *B. verbascifolia* and *P. ramiflora* and fire‐survivors such as *B. coccolobifolia* moderately increased in abundance in the control area (*Δ*_abundance_ = 2.19, 2.51, 2.94), which intensified in the burned area (*Δ*_abundance_ = 5.84, 4.13, 15.43). The NMDS analysis (stress <0.05) showed a clear separation between the fire treatments and greater compositional change over time in the burned plot (Figure 6).

## DISCUSSION

Our study identified a clear distinction between fire‐thriving and fire‐sensitive tree species, using a functional approach and demographic patterns. We found that the functional strategies trees employed to cope with fire (question 1) primarily centered on investment in bark thickness (both outer and inner), wood density in branches and the main trunk, leaf water potential dynamics, leaf water and dry matter content, vegetative phenology, and bud protection. The traits that best‐explained tree mortality and topkill (question 2) were the thickness of both inner and outer bark, and level of protection afforded to sprouting buds. The interaction between the fire‐tolerance responses of the species and the presence of annual fires (question 3) drove significant changes in the horizontal structure and dominance hierarchy of the community, leading to a homogenization of the tree community, favoring fire‐thriving species with conservative strategies related to water balance, drought resistance, and fire tolerance.

### Fire‐response strategies of transitional trees to deal with fire

We found that fire‐response strategies in transitional trees were primarily determined by the investment balance (trade‐off) between inner and outer bark. While on the one hand we found strong trade‐offs, on the other, we found a lot of variability between species, which may have made it difficult to capture the variance of the functional space in a few axes (Díaz et al., 2016). Species with greater investment in outer bark and less in inner bark are better adapted to survive and recruit in fire‐prone environments, making them fire‐thrivers. This trade‐off is an important ecological strategy for the woody components of Cerrado savannas (Scalon et al., 2020), where they frequently experience the effects of fire combined with strong climate seasonality (Coutinho, 1990). The investment in outer or inner bark results in different strategies for dealing with the first‐order effects of fire (Michaletz and Johnson, 2007). Outer bark provides protection against heat transfer to the cambium and prevents physical damage such as tissue necrosis, at the expense of lower light and CO_2_ permeability, limiting stem photosynthesis (Michaletz and Johnson, 2007; Rosell et al., 2014). In contrast, inner bark not only enhances stem photosynthesis but also contributes to water storage (Rosell et al., 2014), increasing stem moisture and acting as a heat barrier (Brando et al., 2012), which is especially advantageous against fire and water deficit during the dry season (Scalon et al., 2020).

Our results also showed that leaf water potential in fire‐sensitive species was more negative post‐fire, suggesting that fire impairs hydraulic function, reducing the xylem capacity to transport water (Thompson et al., 2017; Bär et al., 2019). A reduction in pre‐dawn water potential indicates fire‐induced hydraulic system impairment (Thompson et al., 2017), potentially leading to cavitation and tree mortality over time (Balfour and Midgley, 2006). These findings highlight the need for further studies on the role of fire‐damage‐induced embolism in post‐fire mortality and recovery.

Fire‐survival species exhibited higher wood densities than fire‐thrivers, indicating a growth‐survival trade‐off related to tree architecture, hydraulics, and growth (Pérez‐Harguindeguy et al., 2016). Fire‐thrivers have less‐dense wood due to greater investment in outer bark thickness. Wood density has been shown to be a good predictor of bark densities (inner and outer), which in turn are negatively related to bark thickness (Rosell et al., 2014). High wood density enables trees to compartmentalize fire‐induced wood decay (Romero and Bolker, 2008), reducing tree mortality after fire in transitional forests that have burned at least once (Brando et al., 2012). Although branch wood density is often overlooked, this functional trait plays a crucial role in the mechanical support and stability of the tree canopy (Sterck et al., 2006; He and Deane, 2016). In theory, trunk and branch wood densities are expected to be similar within the same tree because wood density is a phylogenetically conserved trait (Chave et al., 2009), reflecting the genetic regulation of wood production within a species or taxonomic group (Macfarlane, 2020). In our study, we generally observed consistent wood density values in both trunk and branches in most species, except for *Aspidosperma* sp., *M. bella*, and *H. stigonocarpa*.

Although no significant differences were found in specific leaf area (SLA), leaf water content (LWC), or leaf dry matter content (LDMC) across fire tolerance groups, these traits remain important for defining ecological strategies related to the leaf economic spectrum. The fact that these traits do not differ individually is due to the fact that the groups share common trait values and leaf‐related strategies. Our results showed that with regard to leaf economic spectrum and acquisitive strategies, fire‐sensitive species are similar to fire‐survival species, investing more in this strategy compared to fire‐thrivers. Fire‐sensitive species tended to have higher SLA and LWC, a pattern typical of plants in fire‐prone environments (Scalon et al., 2017; Abedi et al., 2022). High SLA and low LDMC (or high LWC) are positively associated with faster‐growth strategy and higher photosynthetic rates (Westoby et al., 2002; Wright et al., 2004). Fast‐growing species often invest less in functional traits for defense, resource conservation, and hydraulic safety (Scalon et al., 2017, 2020; Oliveira et al., 2021), which increases their mortality rates in fire‐prone ecosystems.

Deciduousness emerged as a key trait for fire‐resistant species, likely due to the timing of the experimental burns during the dry season (except for *B. verbascifolia*). This timing favors deciduous trees, which shed their leaves strategically during this period to reduce water stress and consequently avoid leaf tissue necrosis from fire. Additionally, there is some evidence that deciduousness in Cerrado trees is related to deeper rooting depth (Franco et al., 2005). This relationship could further explain species’ higher resistance to fire as their deep root system allows better access to soil water, promoting leaf regrowth at the end of the dry season and after fire events. In that scenario (less severe fire and less water stress), evergreen species would have a competitive advantage due to their greater investment in leaf construction, which acts as an adaptation allowing for better tolerance of leaf scorching under frequent, low intensity regimes (Reich et al., 1997). Deciduous species, having just produced new, less‐resistant leaves, would be more vulnerable (Scalon et al., 2020). Therefore, the current novel fire regime, occurring annually at the peak of the dry season and driven by anthropogenic activities (Pivello et al., 2011), may be detrimental and impact negatively the recovery of evergreen tree species.

Species that were classified as more fire‐sensitive were also associated with lower levels of bud protection and, as previously shown, thinner outer bark. Studies have demonstrated that low or no bud protection is strongly related to higher mortality of woody species in forests and savannas (Charles‐Dominique et al., 2015; Scalon et al., 2020; Chiminazzo et al., 2021, 2023). Bud protection enhances survival by increasing the likelihood of resprouting, though it comes at a high cost in bark thickness and bud reserve (Vesk and Westoby, 2004). However, we also found fire‐thriving species with low levels of bud protection in terms of bark (e.g., *Q. parviflora* and *Q. grandiflora*). A recent study has identified a high density of trichomes covering the buds of *Q. grandiflora* (Chiminazzo et al., 2021), providing an additional protective factor that helps minimize fire damage by maintaining hydration via mucilage secretion. (Werker, 2000; Chiminazzo et al., 2021). Over time, species that lack bud protection and are not fire resistant (they survive but do not resprout) are likely to be excluded from fire‐prone ecosystems (Clarke et al., 2013).

### Key traits driving tree mortality and topkill in a transitional fire‐prone ecosystem

Our results indicate that the key traits explaining topkill and post‐fire mortality are the thickness of inner and outer bark and the level of bud protection. We also found that LDMC and LWC are linked to topkill, while leaf thickness correlates more with the mortality rate. Bark thickness plays a crucial role in preventing heat penetration into the cambium and bud bank (Nolan et al., 2020), which is vital even for large trees to avoid fire damage (Lawes et al., 2011) despite larger height and diameter conferring a survival advantage. For instance, *T. paniculata*, a dominant canopy species, was classified as highly sensitive to fire despite its large size. Our results indicate that bud protection enhanced post‐fire survival and resistance to topkill by allowing for the persistence of aerial and basal bud banks, enabling resprouting after burning and scorching (Vesk and Westoby, 2004). Species with less protection therefore had higher mortality rates and higher levels of topkill. It is important to note, however, that bud protection and resprouting do not guarantee survival because resprouting stems are more vulnerable to subsequent fires, increasing the likelihood of mortality (Hoffmann, 1999; Hoffmann et al., 2009). Thus, with a lower fire frequency, these same species could have a reduced mortality rate. Additionally, leaf traits like water and dry matter content influence the intrinsic flammability of the tree, with higher leaf dry matter content leading to greater topkill risk (Frejaville et al., 2013; Pérez‐Harguindeguy et al., 2016).

Wood density was not shown to be predictive of mortality rate and post‐fire topkill. We believe that this trait is not directly related to fire response, considering that bark traits (OBT and IBT) have a more direct role in savanna species (Rosell et al., 2014). Wood density is usually associated with drought resistance and tree growth (Chave et al., 2009), but it may not be the best predictor of fire survival in savanna species, although it may have a more effective response in forest species (Brando et al., 2012).

While these traits are important, tree mortality and survival after fire also depend on interactions with other abiotic factors (e.g., fire regime, climate, drought, nutrients, and water availability) and biotic pressures (e.g., herbivory and competition) (Enright et al., 2014; Keeley et al., 2019; Nolan et al., 2021). Ultimately, bark thickness and bud protection are critical as the first line of defense and the primary response after fire, respectively. Post‐fire physiological responses, such as foliage necrosis, reduced assimilation, root carbon depletion, carbon starvation, and root death further influence tree survival (see Michaletz et al., 2012; Bär et al., 2019).

### Effects of tree species fire‐tolerance on the transitional tree community

Comparing burned and unburned areas, we found that annual fire significantly influenced community abundance over time, leading to substantial differences in both species composition and structure. These differences are primarily driven by increased tree mortality and canopy opening, which create favorable conditions for the recruitment of new individuals (Oliveras et al., 2014).

Tree species highly sensitive to annual fire, such as *T. paniculata*, *V. haenkeana*, and *X. aromatica*, markedly declined in abundance and are trending toward exclusion from the community. These species typically thrive in the absence of fire. The introduction of annual fire substantially altered the relationship between dominant and subordinate species of the community, increasing both mortality and recruitment. Fire acts as a strong environmental filter for woody species (Enright et al., 2015), favoring species with functional traits that promote resistance or recovery after disturbance (Bond and Keeley, 2005; Oliveras et al., 2014). Consistent with other studies (Cavender‐Bares and Reich, 2012; Pellegrini et al., 2023), fire negatively impacted the abundance of species with acquisitive strategies, and areas exposed to frequent fire for extended periods (<5 years) tend to be dominated by deciduous species with low foliar nitrogen concentration (Pellegrini et al., 2021). This shift in species composition directly affects ecosystem functions, such as carbon balance and nutrient cycling, by altering functional trait distribution (Mitchell et al., 2021). An altered fire regime not only changes community structure (Hoffmann and Moreira, 2002), but also directly impacts the balance of functional groups (Scalon et al., 2020), leading to taxonomic and functional homogenization (Silva et al., 2018; Weeks et al., 2023).

## CONCLUSIONS

The annual fire regime applied in this study may have considerably affected species mortality, underscoring the need for a broader exploration of different fire frequencies, intensities, and different vegetation types (Pivello et al., 2021). This study highlights how a range of different functional traits, both individually and collectively, shape the fire tolerance of transitional tree species under an altered fire regime. We identified key indicators that reveal how certain functional groups are more impacted than others facing a novel fire regime, influencing community structure. Future research should expand this framework to include additional functional traits, particularly root traits (Cusack et al., 2021), which play an important role in the survival strategies of species in fire‐prone ecosystems.

## AUTHOR CONTRIBUTIONS

I.O.M. and W.J.A.C. designed the study; I.O.M. got the funding and supervised different stages of the study; F.C.O., W.J.A.C., F.N.R., and M.M. did the fieldwork; W.J.A.C. organized the database; W.J.A.C. and F.N.R analyzed the data; M.L.F. and M.A.C helped with field logistics; W.J.A.C. wrote the first draft; I.O.M., M.M., M.A.C., and F.N.R. discussed and revised drafts.

## Supporting information


**Appendix S1**. Figures and tables from PCA of quantitative and qualitative variables.

**Appendix S2**. Figures and statistics relating to Kruskal–Wallis tests comparing functional traits between the fire‐tolerance groups.

**Appendix S3**. Figures and statistics for mixed linear model to test for differences in the change in abundance over time between species in the burned and unburned plots.

## Data Availability

The data are available in the Zenodo digital repository: 10.5281/zenodo.15275872 (https://zenodo.org/records/15275872).
