# Supplementary material for: Deciphering fire tolerance of trees at the Amazonia–Cerrado transition by trait‐based approach: Implications from species to communities
Source: Am J Bot. 2025 Jul 3;112(10):e70066. doi: 10.1002/ajb2.70066 (PMC12572680; doi:10.1002/ajb2.70066)
Supplement: Supplementary file 1 — Appendix S1. Figures and tables from PCA of quantitative and qualitative variables. [file AJB2-112-e70066-s001.docx]

Cruz et al.—American Journal of Botany 2025—Appendix S1

**Appendix S1**. Figures and tables from PCA of quantitative and qualitative variables.


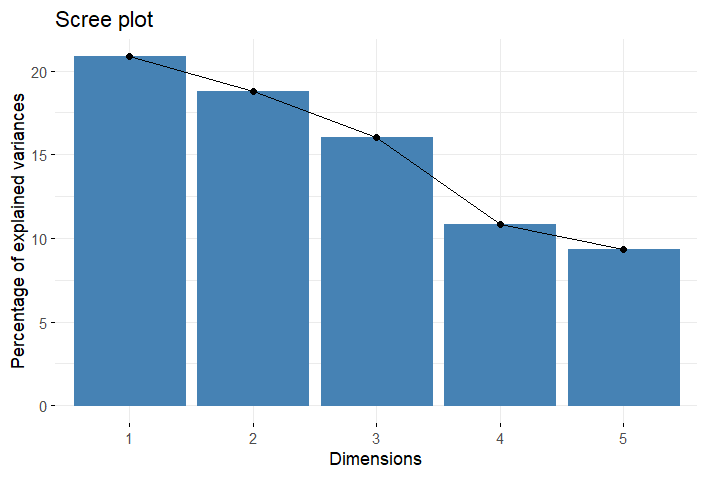


**Figure S1.** Percentage of variation explained by each dimension of PCA.


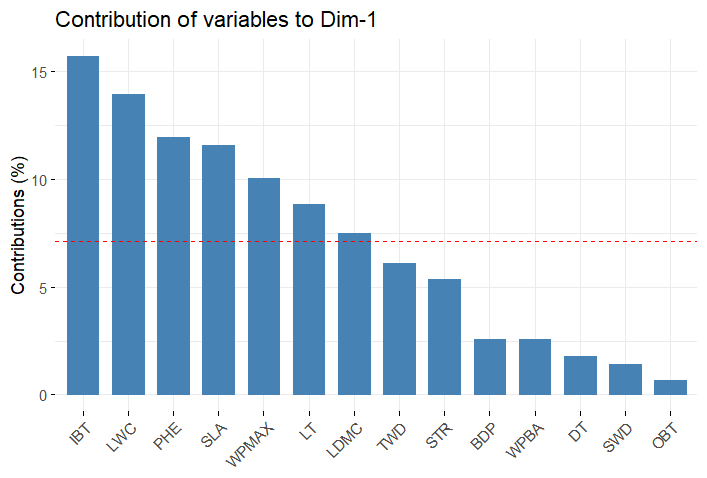


**Figure S2.** Bar plot showing the percentage contribution of variables to Dimension 1 from PCA. SLA = specific leaf area, LDMC = leaf dry matter content, LT = leaf thickness, LWC = leaf water content, WPMAX = maximum leaf water potential, WPBA = change in leaf water potential, OBT = outer bark thickness, IBT = inner bark thickness, DT = bark density, SWD = stem wood density, TWD = twig wood density, PHE = vegetative phenology, BDP = bud protection, STR = fire-tolerance strategy.


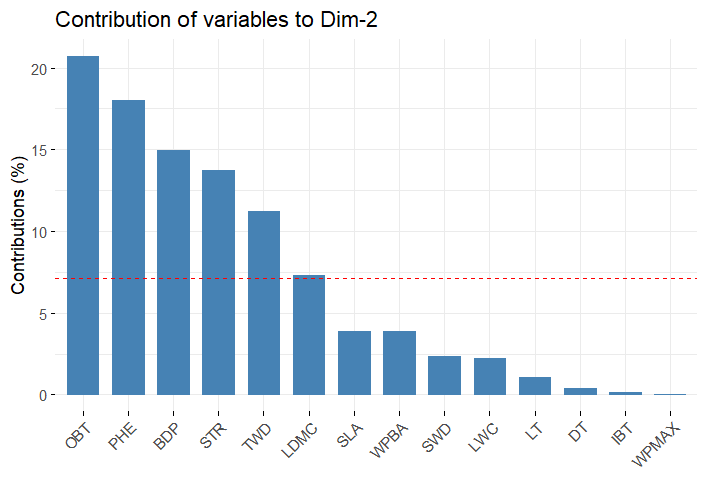


**Figure S3.** Bar plot showing percentage contribution of variables to Dimension 2 from PCA. SLA = specific leaf area, LDMC = leaf dry matter content, LT = leaf thickness, LWC = leaf water content, WPMAX = maximum leaf water potential, WPBA = change in leaf water potential, OBT = outer bark thickness, IBT = inner bark thickness, DT = bark density, SWD = stem wood density, TWD = twig wood density, PHE = vegetative phenology, BDP = bud protection, STR = fire-tolerance strategy.


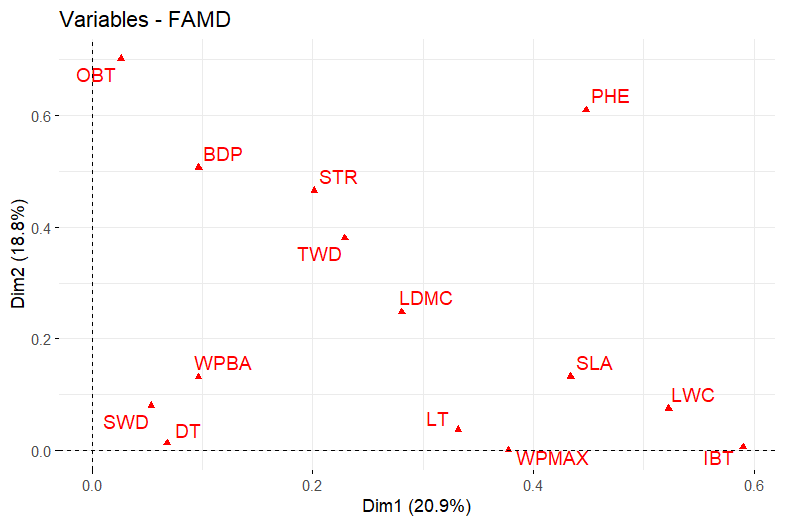


**Figure S4**. Correlation between all the variables, both quantitative and qualitative, from the PCA. SLA = specific leaf area, LDMC = leaf dry matter content, LT = leaf thickness, LWC = leaf water content, WPMAX = maximum leaf water potential, WPBA = change in leaf water potential, OBT = outer bark thickness, IBT = inner bark thickness, DT = bark density, SWD = stem wood density, TWD = twig wood density, PHE = vegetative phenology, BDP = bud protection, STR = fire-tolerance strategy.

**Table S1.** Eigenvalues for PCA for all functional traits for the first five dimensions. SLA = specific leaf area, LDMC = leaf dry matter content, LT = leaf thickness, LWC = leaf water content, *Ψ*_IMAX_ = maximum leaf water potential, *Ψ*_IΔ_ = change in leaf water potential, OBT = outer bark thickness, IBT = inner bark thickness, BD = bark density, SWD = stem wood density, TWD = twig wood density, PHE = vegetative phenology, BP = bud protection.

| Trait | Dim1 | Dim2 | Dim3 | Dim4 | Dim5 |
| --- | --- | --- | --- | --- | --- |
| SLA (mm^2^ mg^–1^) | 0.4339 | 0.1316 | 0.1703 | 0.1354 | 0.0012 |
| LDMC (%) | 0.2807 | 0.2479 | 0.05381 | 0.07949 | 0.0178 |
| LT (mm) | 0.3320 | 0.0369 | 0.1291 | 0.0285 | 0.0107 |
| LWC (%) | 0.5227 | 0.0749 | 0.0013 | 0.0622 | 0.0031 |
| *Ψ*_IMAX_ (MPa) | 0.3773 | 0.0001 | 0.2345 | 0.0668 | 0.0071 |
| *Ψ*_IΔ_ (MPa) | 0.0961 | 0.1315 | 0.0205 | 0.0151 | 0.5198 |
| OBT (mm) | 0.0258 | 0.7016 | 0.0046 | 0.0826 | 0.0188 |
| IBT (mm) | 0.5902 | 0.0056 | 0.0302 | 0.0694 | 0.0218 |
| BD (g cm^–3^) | 0.0677 | 0.0128 | 0.7013 | 0.0002 | 0.0504 |
| SWD (g cm^–3^) | 0.0534 | 0.0801 | 0.6842 | 0.0071 | 0.0167 |
| TWD (g cm^–3^) | 0.2289 | 0.3801 | 0.0611 | 0.0657 | 0.0858 |
| PHE | 0.4477 | 0.6095 | 0.2228 | 0.1749 | 0.2174 |
| BP | 0.0966 | 0.5067 | 0.5014 | 0.8206 | 0.3278 |
