# Supplementary material for: Deciphering fire tolerance of trees at the Amazonia–Cerrado transition by trait‐based approach: Implications from species to communities
Source: Am J Bot. 2025 Jul 3;112(10):e70066. doi: 10.1002/ajb2.70066 (PMC12572680; doi:10.1002/ajb2.70066)
Supplement: Supplementary file 2 — Appendix S2. Figures and statistics relating to Kruskal–Wallis tests comparing functional traits between the fire‐tolerance groups. [file AJB2-112-e70066-s002.docx]

Cruz et al. - American Journal of Botany 2025 - Appendix S2

**Appendix S2**. Figures and statistics relating to Kruskal–Wallis tests comparing functional traits between the fire-tolerance groups.


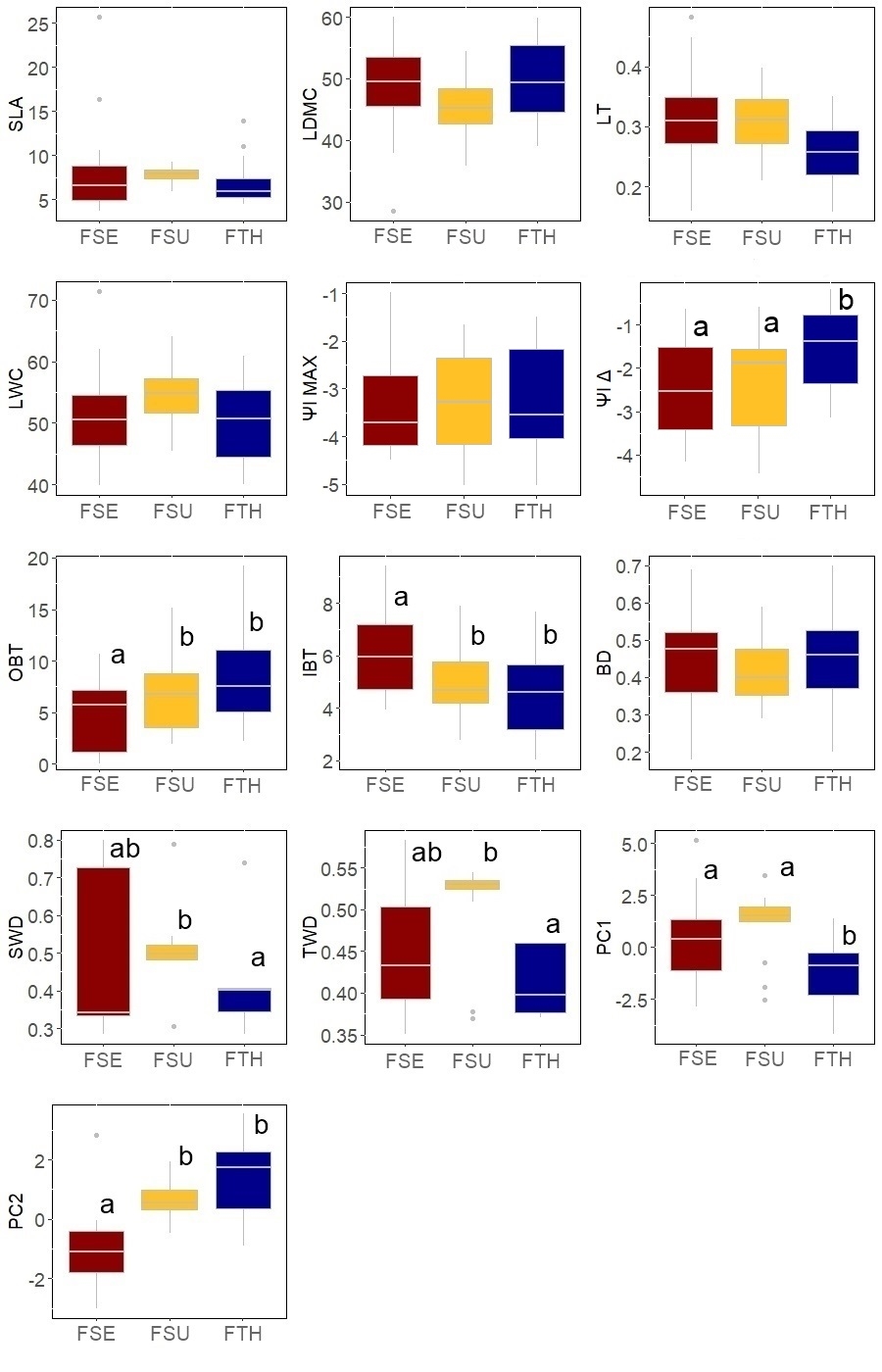


**Figure S5.** Comparison of all functional traits and PCA axis scores between different fire-tolerance strategies in transitional savannah tree species. Different letters denote statistical difference according to Kruskal–Wallis tests. FSE = fire-sensitive; FSU = fire-survival, FTH = fire-thrivers. SLA = specific leaf area, LDMC = leaf dry matter content, LT = leaf thickness, LWC = leaf water content, *Ψ*_IMAX_ = maximum leaf water potential, *Ψ*_IΔ_ = change in leaf water potential, OBT = outer bark thickness, IBT = inner bark thickness, BD = bark density, SWD = stem wood density, TWD = twig wood density, PC 1 and 2 = scores for PCA axis.

**Table S2.** Statistics for Kruskal–Wallis test comparing functional traits and different fire tolerance strategies. SLA = specific leaf area, LDMC = leaf dry matter content, LT = leaf thickness, LWC = leaf water content, *Ψ*_IMAX_ = maximum leaf water potential, *Ψ*_IΔ_ = change in leaf water potential, OBT = outer bark thickness, IBT = inner bark thickness, BD = bark density, SWD = stem wood density, TWD = twig wood density, PC 1 and 2 = scores for PCA axis.

| Trait | df | *χ*^2^ | *P* |
| --- | --- | --- | --- |
| SLA | 2 | 4.4592 | 0.107 |
| LDMC | 2 | 4.938 | 0.084 |
| LT | 2 | 3.694 | 0.157 |
| LWC | 2 | 4.938 | 0.084 |
| *Ψ*_IMAX_ | 2 | 0.366 | 0.832 |
| *Ψ*_IΔ_ | 2 | 3.900 | 0.014 |
| OBT | 2 | 4.204 | 0.012 |
| IBT | 2 | 10.305 | 0.005 |
| BD | 2 | 1.656 | 0.436 |
| SWD | 2 | 6.286 | 0.043 |
| TWD | 2 | 8.273 | 0.015 |
| PC1 | 2 | 13.915 | 0.001 |
| PC2 | 2 | 22.519 | <0.001 |
