# Supplementary material for: Deciphering fire tolerance of trees at the Amazonia–Cerrado transition by trait‐based approach: Implications from species to communities
Source: Am J Bot. 2025 Jul 3;112(10):e70066. doi: 10.1002/ajb2.70066 (PMC12572680; doi:10.1002/ajb2.70066)
Supplement: Supplementary file 3 — Appendix S3. Figures and statistics for mixed linear model to test for differences in the change in abundance over time between species in the burned and unburned plots. [file AJB2-112-e70066-s003.docx]

Cruz et al. - American Journal of Botany 2025 - Appendix S3

**Appendix S3**. Figures and statistics for mixed linear model to test for differences in the change in abundance over time between species in the burned and unburned plots.

**Table S3.** Statistics for mixed linear model comparing changes in species abundance (ΔAbundance) between the beginning and end of monitoring of the burned and unburned areas. Groups = random factor grouping unit, Name = model terms for which variances and correlations were estimated within each group, Corr = correlation.

| Random effects |  |  |  |  |  |
| --- | --- | --- | --- | --- | --- |
| Groups | Name | Variance | SD | Corr |  |
| Species | Unburned | 0.1339 | 0.3659 |  |  |
|  | Burned | 0.8161 | 0.9034 | -0.93 |  |
| Residual |  | 0.6496 | 0.8060 |  |  |
|  |  |  |  |  |  |
| Fixed effects |  |  |  |  |  |
|  | Estimate | SE | df | *t* | *P* |
| Unburned | 0.2293 | 0.1032 | 13.9998 | 2.221 | 0.0433 |
| Burned | -0.3813 | 0.2406 | 13.9998 | -1.585 | 0.0135 |


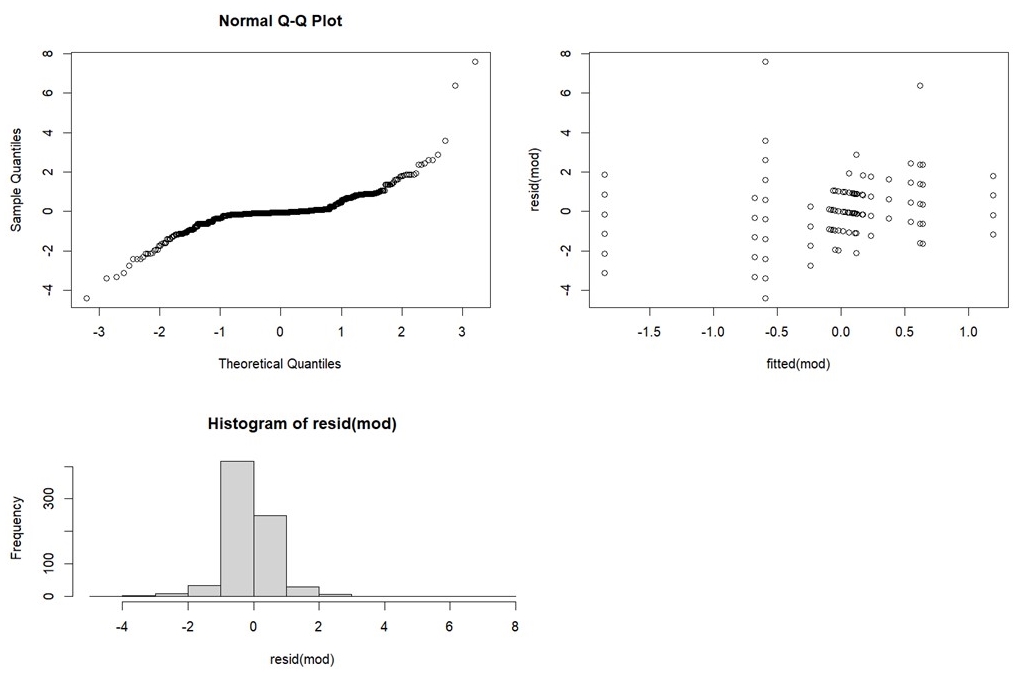


**Figure S6.** Histogram and Q-Q plot of the residuals of the changes in species abundance (ΔAbundance) between the beginning and end of monitoring of the burned and the unburned areas determined by mixed linear model.
